# Diagnosability of Keratoconus Using Deep Learning With Placido Disk-Based Corneal Topography

**DOI:** 10.3389/fmed.2021.724902

**Published:** 2021-10-04

**Authors:** Kazutaka Kamiya, Yuji Ayatsuka, Yudai Kato, Nobuyuki Shoji, Yosai Mori, Kazunori Miyata

**Affiliations:** ^1^Visual Physiology, School of Allied Health Sciences, Kitasato University, Kanagawa, Japan; ^2^Cresco Ltd., Tokyo, Japan; ^3^Department of Ophthalmology, School of Medicine, Kitasato University, Kanagawa, Japan; ^4^Department of Ophthalmology, Miyata Eye Hospital, Miyazaki, Japan

**Keywords:** deep learning, keratoconus, diagnosis, accuracy, corneal topography

## Abstract

**Purpose:** Placido disk-based corneal topography is still most commonly used in daily practice. This study was aimed to evaluate the diagnosability of keratoconus using deep learning of a color-coded map with Placido disk-based corneal topography.

**Methods:** We retrospectively examined 179 keratoconic eyes [Grade 1 (54 eyes), 2 (52 eyes), 3 (23 eyes), and 4 (50 eyes), according to the Amsler-Krumeich classification], and 170 age-matched healthy eyes, with good quality images of corneal topography measured with a Placido disk corneal topographer (TMS-4^TM^, Tomey). Using deep learning of a color-coded map, we evaluated the diagnostic accuracy, sensitivity, and specificity, for keratoconus screening and staging tests, in these eyes.

**Results:** Deep learning of color-coded maps exhibited an accuracy of 0.966 (sensitivity 0.988, specificity 0.944) in discriminating keratoconus from normal eyes. It also exhibited an accuracy of 0.785 (0.911 for Grade 1, 0.868 for Grade 2, 0.920 for Grade 3, and 0.905 for Grade 4) in classifying the stage. The area under the curve value was 0.997, 0.955, 0.899, 0.888, and 0.943 as Grade 0 (normal) to 4 grading tests, respectively.

**Conclusions:** Deep learning using color-coded maps with conventional corneal topography effectively distinguishes between keratoconus and normal eyes and classifies the grade of the disease, indicating that this will become an aid for enhancing the diagnosis and staging ability of keratoconus in a clinical setting.

## Introduction

Keratoconus has been widely recognized as a progressive disease characterized by anterior bulging and local thinning of the cornea. Based on the fact that keratoconic patients tend to have high myopic astigmatism, the percentage of such patients among all candidates for corneal refractive surgery has shown to be relatively high. Therefore, it is clinically essential to effectively exclude keratoconus among refractive surgery candidates to prevent the post-operative occurrence of iatrogenic keratectasia.

In previous studies, simple multi-layer neural networks, support vector machines, or decision trees were applied to machine learning for keratoconus detection. We assume that the use of a convolutional neural network has advantages over other machine learning methods, since a convolutional neural network can directly extract the morphological characteristics from the obtained images without preliminary learning, and subsequently provide a higher classification precision, especially in the field of image recognition.

We previously reported that deep learning using multiple color-coded maps obtained from the anterior segment optical coherence tomography (OCT) was effective not only for the screening of keratoconus, but also for the grade classification (1). Placido disk-based corneal topography provides a high sensitivity and specificity to discriminate keratoconus from normal eyes, and is still widely used especially at private eye clinics in a clinical setting. Although corneal topography only examines the anterior corneal surface, it is still most commonly used in daily practice. However, deep learning technique has so far not been fully elucidated for keratoconus detection using Placido disk-based corneal topography. Moreover, this technique has not been applied for the keratoconus staging capability using corneal topography. It may be clinically meaningful not only as a pre-operative screening test of refractive surgery candidates, but also as a staging test for understanding the severity of the disease, especially in consideration of high prevalence of such devices. The goal of the present study is to evaluate the diagnostic capability of deep learning using conventional corneal topography, in terms of the disease screening and the stage classification.

## Methods

### Study Population

We registered the study protocol with the University Hospital Medical Information Network Clinical Trial Registry (000040128). A total of 349 eyes with good quality images of corneal topography measured with a Placido disk corneal topographer (TMS-4^TM^, Tomey, Aichi, Japan) were included in this case series. Multiple corneal specialists diagnosed keratoconus with distinctive features (e.g., corneal color-coded map with asymmetric bow-tie pattern with or without skewed axes), and at least one keratoconus sign (e.g., stromal thinning, conical bulging, Fleischer ring, Vogt striae, or apical scar) (2). We utilized the Amsler-Krumeich classification to evaluate the grade of the disease [Grade 1 (54 eyes), 2 (52 eyes), 3 (23 eyes), and 4 (50 eyes)] (3). As a control group we examined 170 eyes in subjects with normal ocular findings applying for a contact lens fitting or for a refractive surgery consultation, who had a refractive error of <6 diopters (D) as well as astigmatism of <3 D. We asked the patients who wore rigid and soft contact lenses to stop wearing them for 3 and 2 weeks, respectively. This review was approved by the Institutional Review Board of Miyata Eye Hospital (CS-315), and followed the tenets of the Declaration of Helsinki. The Institutional Review Board waived the requirement for informed consent for this retrospective study.

### Placido Disk-Based Corneal Topography

We performed corneal topography using a Placido disk-based corneal topographer (TMS-4™, Tomey Corporation, Nagoya, Japan). Patients were asked to blink just before starting measurements. We obtained an absolute topography map (9.0–101.5 diopter (D), 5 D step), in accordance with the manufacturer's instructions, because this scale is widely used in daily practice. We acquired at least three topographic images with this corneal topographer. We manually excluded poor-quality data (e.g., blinking artifacts, or poor detections of topographic images), and selected one topographic map with a high image quality.

### Deep Learning

Detailed methods for deep learning were described previously (1). In brief, we exported the data of a single image by taking a screenshot, and stored it in a lossless compression format such as PNG. We excluded the color-scaled bar for this image analysis. We made one classifier for the Placido disk-based color-coded map. We utilized an open source deep learning platform (PyTorch) for deep learning with a VGG-16 network model. This model has been pre-trained by 3,390 color-coded map images that were composed of anterior and posterior elevation, anterior and posterior curvature, total refractive power, and pachymetry maps obtained with an anterior segment OCT (CASIA2^TM^, Tomey Corporation, Nagoya, Japan) (1). Each input image without the color-scaled bar was resized to 224-by-224 pixels without deformation. The output (one value of 0–4) can be mapped to the grades (including normal eyes). “Normal” is represented as “0,” and grades 1, 2, 3, and 4 are denoted as “1,” “2,” “3,” and “4” in teaching data. Each network classifies an image into 0–4. The output value of neural network for an image is a real number, so that we aligned it to the nearest integer value to interpret. For example, if the output value is “2.67,” it is interpreted as “3” (classified as Grade 3). A total of 349 eyes were split into seven groups (49 or 50 eyes in each group) based on our preliminary evaluation. We applied 7-fold cross-validation with validation and test set to increase the reliability of the accuracy outcomes of the classifier. For each fold, five of seven sets were used for training and one of the remaining two sets was used for validation, finally the model was tested with the remaining one set after the training finished (training set: validation set: test set = 5:1:1). We calculated the sensitivity, the specificity, and the accuracy as follows: sensitivity = true positive / (true positive + false negative), specificity = true negative/(true negative + false positive), and accuracy = (true positive + true negative)/(true positive + false positive + true negative + false negative). We also calculated the receiver operating characteristic curve and the area under the curve (AUC) as the area under the cumulative distribution function of the sensitivity on the y-axis vs. the cumulative distribution function of (1- specificity) on the x-axis, using a statistical software (Bellcurve for Excel, Social Survey Research Information Co, Ltd., Tokyo, Japan).

## Results

**Figure 1:**
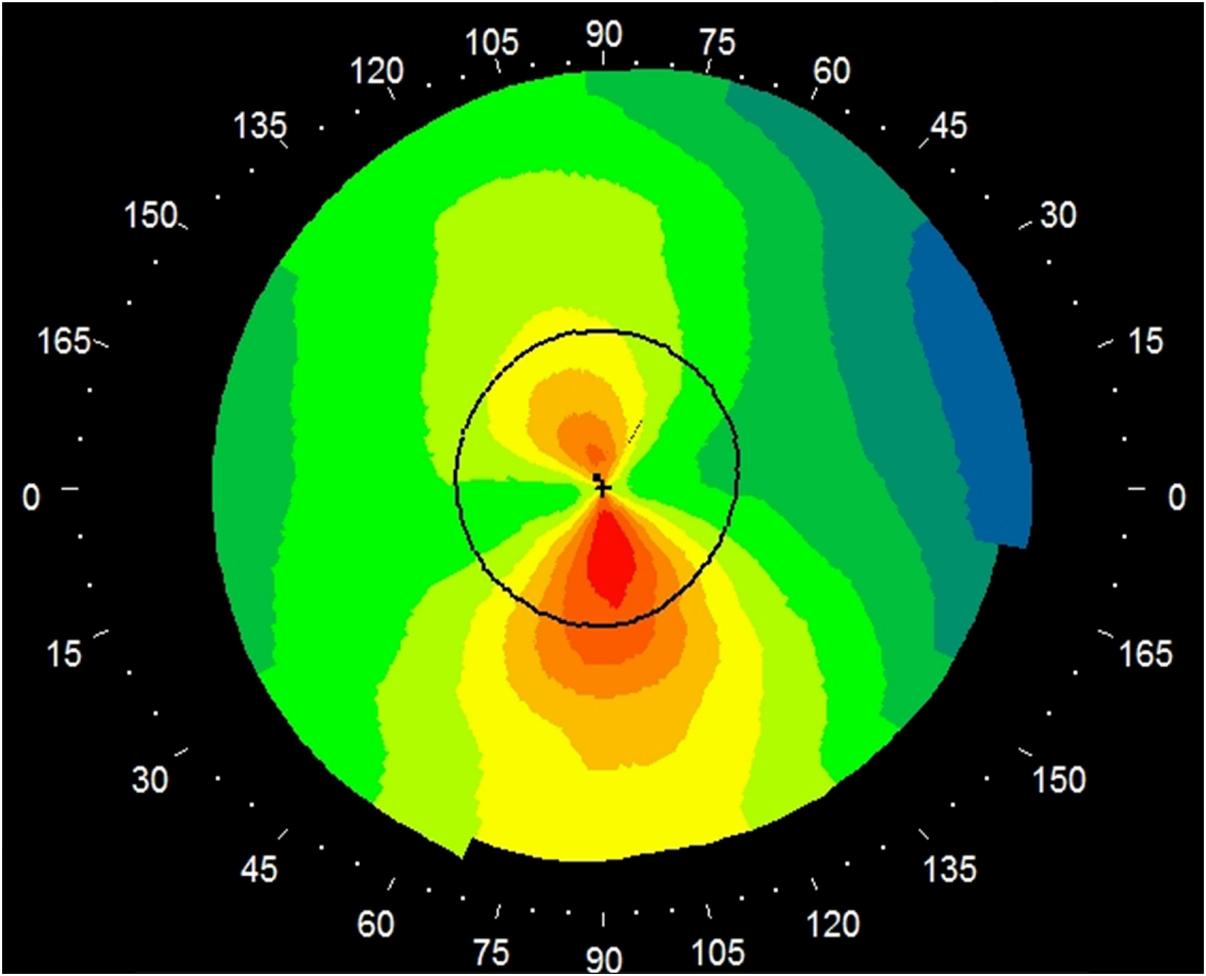
A representative image of a single color-coded map (absolute axial map) by the Placido disk-based corneal topography. A color-scale bar was eliminated for deep learning.

**Figure 2:**
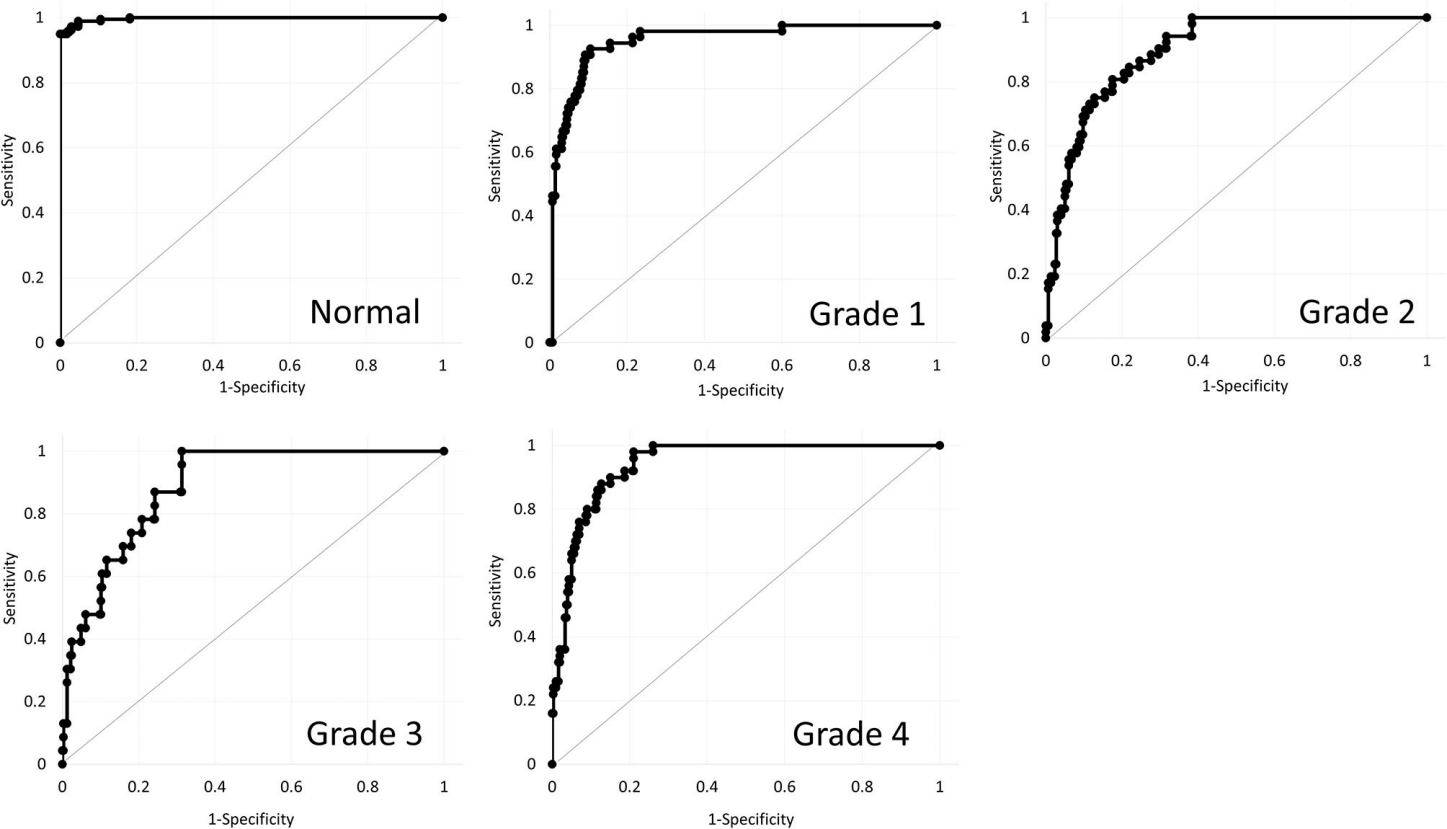
Receiver operating characteristic curves of classifying normal and Grade 1 to 4 keratoconic eyes using deep learning.

Table 1 shows the patient demographics of the study population. Figure 1 shows a representative color-coded map measured with the Placido disk-based corneal topography. Table 2 shows the output data of deep learning of single color-coded maps with corneal topography in terms of the grade classification for test data sets. Table 3 shows the sensitivity, the specificity, and the accuracy of deep learning of a single color-coded map with corneal topography in the grade classification according to the Amsler-Krumeich classification. Deep learning of the color-coded map exhibited an accuracy of 0.966 (sensitivity 0.988, specificity 0.944), in discriminating keratoconus from normal cornea. It also exhibited an overall accuracy of 0.785 (sensitivity 0.611, specificity 0.966 for Grade 1, sensitivity 0.615, specificity 0.912 for Grade 2, sensitivity 0.281, specificity 0.957 for Grade 3, and sensitivity 0.640, specificity 0.950 for Grade 4) in classifying the stage of the disease. Figure 2 shows the receiver operating characteristic curves of classifying normal and Grade 1 to 4 keratoconic eyes, and the AUC value was 0.997, 0.955, 0.899, 0.888, and 0.943 as Grade 0 (normal) to 4 grading tests, respectively.

**Table 1:** The demographics of the study population according to the Amsler-Krumeich classification.

**Characteristic**	**Control**	**Grade 1**	**Grade 2**	**Grade 3**	**Grade 4**
Age (years)	36.9 ± 12.5	34.1 ± 18.0	35.4 ± 14.1	35.4 ± 15.0	39.4 ± 14.6
LogMAR UCVA	0.76 ± 0.66	0.48 ± 0.55	0.94 ± 0.60	0.91 ± 0.56	1.27 ± 0.58
LogMAR BSCVA	−0.17 ± 0.04	−0.03 ± 0.12	0.15 ± 0.32	0.23 ± 0.32	0.46 ± 0.38
Manifest sphere (D)	−3.07 ± 3.44	−2.32 ± 3.52	−4.74 ± 4.63	−4.23 ± 3.99	−4.67 ± 5.43
Manifest cylinder (D)	−0.67 ± 0.73	−2.10 ± 1.77	−3.40 ± 1.86	−3.24 ± 1.55	−2.68 ± 2.61
Flat keratomtry (D)	43.1 ± 1.2	43.2 ± 1.7	46.2 ± 3.0	49.8 ± 4.7	52.6 ± 4.6
Steep keratometry (D)	44.6 ± 1.5	46.3 ± 2.1	51.0 ± 3.8	55.7 ± 5.0	58.8 ± 6.4

*LogMAR, logarithm of the minimal angle of resolution; UCVA, uncorrected visual acuity; BSCVA, best spectacle corrected visual acuity; D, diopter*.

**Table 2:** The output data for test sets of deep learning in the grade classification according to the Amsler-Krumeich classification.

	**Output of Convolutional Neural Network**					
**Actual Category**	**Normal**	**G1**	**G2**	**G3**	**G4**	**Total**
Normal	168	2	0	0	0	170
G1	9	33	11	0	1	54
G2	0	6	32	8	6	52
G3	0	1	5	9	8	23
G4	1	1	10	6	32	50

**Table 3:** The sensitivity, the specificity, and the accuracy of deep learning in the grade classification according to the Amsler-Krumeich classification.

**Category**	**True positive**	**True negative**	**False negative**	**False positive**	**Sensitivity**	**Specificity**	**Accuracy**
Normal	168	169	2	10	0.988	0.944	0.966
G1	33	285	21	10	0.611	0.966	0.911
G2	32	271	20	26	0.615	0.912	0.868
G3	9	312	14	14	0.281	0.957	0.920
G4	32	284	18	15	0.640	0.950	0.905
Total							0.785

## Discussion

In the current study, our findings demonstrated that deep learning of a single color-coded map with a corneal topographer will be beneficial not only for the disease screening, but also for the stage classification. To our knowledge, this is the first study to investigate both the diagnostic and disease staging capability of deep learning of a single image with conventional Placido disk-based corneal topography. In terms of simple keratoconus detection, Kuo et al. compared deep learning algorithms to detect keratoconus on the basis of corneal topography, showing that the accuracy was 0.931, 0.931, and 0.958, when using the VGG16, InceptionV3, ResNet152 models, respectively. However, this study was not age-matched with a significant difference in age between the groups, and the numbers of the subjects (94 and 84) were still limited in the keratoconus and control groups, respectively (4). Since it is still difficult to differentiate keratoconus from normal cornea in daily practice, when using only slit-lamp examination, we assume that it will become an aid as a screening and staging test for keratoconus, especially in consideration of the higher prevalence of corneal topography in a clinical setting.

So far many studies on keratoconus detection have been conducted using machine learning methods. However, most studies have just utilized topographic or tomographic numeric indices that can grasp the overall corneal shape, but this hides the spatial gradients and distributions of the corneal curvature. In recent years, several studies on keratoconus detection using deep learning have been reported (1, 5–9). Dos Santos et al. stated that a custom neural network architecture could segment both healthy and keratoconus images with high accuracy (5). Abdelmotaal et al. demonstrated, using color-coded Scheimpflug images, that a convolutional neural network classified four map-selectable display images with average accuracies of 0.983 and 0.958 for the training and test sets, respectively (7). Elsawy et al. developed the multi-disease deep learning diagnostic algorithm providing an F1 score >0.90 for keratoconus detection using the As-OCT images (8). Feng et al. proposed an end-to-end deep learning approach utilizing raw data obtained by the Pentacam system for keratoconus and subclinical keratoconus detection (9). Chen et al. also showed that convolutional neural network provides excellent performance for keratoconus detection and grading classification using the axial map, anterior and posterior elevation map, and pachymetry maps obtained by the Scheimpflug camera (10). We assume that the use of a color-coded map has advantages over that of numeric values for machine learning, because it can bring us a larger amount of anterior corneal curvature information than these numeric values. These findings were in agreement with our current findings using corneal topography. Our study is somewhat different from their study in that we assessed the stage-classification capability of the disease, which is clinically meaningful to determine the surgical indication, as well as to predict the visual prognosis of such keratoconic patients, and that adopt a cross-validation with validation and test set to increase the reliability of the accuracy outcomes of the classifier.

In the current study, the overall accuracy using a single map with Placido disk-based corneal topography was slightly lower than that using multiple maps with the OCT, in terms of the keratoconus screening (vs. 0.991) and the keratoconus staging (vs. 0.874) (1). Moreover, even when we compared the same single absolute map of anterior corneal curvature between the two instruments, the accuracy using the corneal topography was slightly lower than that using the OCT (vs. 0.976). We previously reported, using the rotating Scheimpflug tomography, that the cases of lower staging had a larger area under the receiver operating characteristic curve in the posterior elevation differences than in the anterior elevation differences (11), and that the accuracy of various elevation, pachymetry, and keratometry indices was overall high, but that posterior and anterior elevation differences were the most effective parameters for the diagnosis of keratoconus (12). These findings highlight the importance of the corneal posterior information as a diagnostic ability using the corneal tomographer. Based on our current and previous findings, we can detect keratoconus only using the Placido-based corneal topography in almost all eyes, but the use of OCT-based or Scheimpflug-based corneal tomography is still recommended to further improve the diagnostic accuracy, especially for the early diagnosis of keratoconus.

Our limitations to this study are as follows: Firstly, we only used normal corneas as a control group, and that we did not include other corneal disorders, such as forme fruste keratoconus, subclinical keratoconus, or post-keratoplasty eyes. Accordingly, we cannot refute the possibility that the disease category and the inclusion criteria might influence the diagnosability of keratoconus. Secondly, we did not totally eliminate the effect of rigid or soft contact lenses on corneal topographic measurements. Although we asked the patients to stop wearing rigid gas permeable lenses and soft contact lenses for 3 and 2 weeks, respectively, before this evaluation, it is clinically difficult for such patients to stop wearing contact lenses for a long period of time, in consideration of their daily life activities. Thirdly, it is still difficult to accurately diagnose keratoconus by ophthalmologists, and its diagnosis can be influenced not only by the definition but also by the severity of the disease. We still need to have ophthalmologists read the same dataset and compare the performance of this model with the ophthalmologists' readings. Fourthly, we used healthy eyes with astigmatism of <3 D as a control group, in accordance with previous studies on keratoconus detection. Therefore, we did not guarantee that healthy eyes with a large amount of astigmatism could be classified as normal eyes using this network. A further research in another population is still necessary to clarify this point.

## Conclusions

In summary, our findings may support the view that deep learning of a single color-coded map with a conventional corneal topographer is clinically helpful not only for keratoconus screening, but also for stage classification. We assume that it will also become an aid for keratoconus detection using a conventional topography in daily practice. We await a further external validation using another study population to confirm the authenticity of our results.

## Data Availability Statement

The original contributions presented in the study are included in the article/supplementary material, further inquiries can be directed to the corresponding author.

## Ethics Statement

The studies involving human participants were reviewed and approved by Institutional Review Board of Miyata Eye Hospital (CS-315). Written informed consent from the participants' legal guardian/next of kin was not required to participate in this study in accordance with the national legislation and the institutional requirements.

## Author Contributions

KK, NS, and KM were involved in the design and conducted the study. KK, YA, YK, and YM were involved in collection, management, analysis, and interpretation of data. KK, YA, YK, NS, YM, and KM were involved in preparation, review, and final approval of the manuscript. All authors contributed to the article and approved the submitted version.

## Funding

This work was in part supported by Grants-in-Aid for Scientific Research (Grant Number 21K09706).

## Conflict of Interest

YA and YK are employed by Cresco Co Ltd. The remaining authors declare that the research was conducted in the absence of any commercial or financial relationships that could be construed as a potential conflict of interest.

## Publisher's Note

All claims expressed in this article are solely those of the authors and do not necessarily represent those of their affiliated organizations, or those of the publisher, the editors and the reviewers. Any product that may be evaluated in this article, or claim that may be made by its manufacturer, is not guaranteed or endorsed by the publisher.
